# Antibody response to double SARS-CoV-2 mRNA vaccination in Japanese kidney transplant recipients

**DOI:** 10.1038/s41598-022-10510-7

**Published:** 2022-04-27

**Authors:** Kumiko Fujieda, Akihito Tanaka, Ryosuke Kikuchi, Nami Takai, Shoji Saito, Yoshinari Yasuda, Takashi Fujita, Masashi Kato, Kazuhiro Furuhashi, Shoichi Maruyama

**Affiliations:** 1 Department of Nephrology, Nagoya University Hospital, Tsurumaicho, 65, Showa Ward, Nagoya, Aichi Japan; 2 Department of Medical Technique, Nagoya University Hospital, Nagoya, Aichi Japan; 3 Department of Nursing, Nagoya University Hospital, Nagoya, Aichi Japan; 4 Department of Urology, Nagoya University Graduate School of Medicine, Nagoya, Aichi Japan; 5 Department of Nephrology, Nagoya University Graduate School of Medicine, Nagoya, Japan

**Keywords:** Nephrology, Viral infection

## Abstract

Immunocompromised patients, especially those who undergo kidney transplantation, have lower antibody levels following SARS-CoV-2 mRNA vaccination. The situation of transplant treatment, such as transplant source and immunosuppressive drugs, is different in Japan than that in other countries. Therefore, it is necessary to clarify whether antibody acquisition rates differ between Japan and other countries. This retrospective study included patients with post-kidney transplant who were attending at the Nagoya University Hospital. The anti-SARS-CoV-2 IgG antibody titers were measured between 3 weeks and 3 months after vaccination. Seventy-three patients (45 men and 28 women) were included. Of these, 23 (31.5%) showed antibody presence, and the rates of antibody acquisition were very low than those in the control group (100.0% vs. 31.5%, *P* < 0.05). Antibody acquisition rates were associated with body mass index (odds ratio [OR]: 1.21, 95% confidence interval [CI]: 1.04–1.39, *P* < 0.05) and the duration between transplantation and vaccination (OR: 1.01, 95% CI: 1.00–1.02, *P* < 0.05). The immunosuppressive drugs used were: prednisolone in all cases, tacrolimus in 89.0%, cyclosporine in 9.6%, and mofetil mycophenolate in 97.3%. None of the patients were excluded from receiving two doses of the vaccine due to adverse effects. The study indicated that vaccination-induced antibody acquisition rates against SARS-CoV-2 were extremely low in Japanese patients who underwent post-kidney transplantation. Thus, despite two doses of vaccination, it is necessary to closely monitor infection control in such patients.

## Introduction

The COVID-19 pandemic that started in 2019 has killed many patients^1^. It has been reported that immunocompromised patients, such as those with underlying diseases, are at a high risk for developing severe disease and need to be careful about infection^2^. The SARS-CoV-2 mRNA vaccines have shown high antibody acquisition rates in healthy individuals, indicating that they are useful in preventing infections and severe diseases^3,4^. However, immunocompromised patients, especially those after kidney transplantation, have low rates of antibody acquisition after SARS-CoV-2 mRNA vaccination than immunologically healthy individuals^5,6^. The antibody acquisition rates are higher with two doses of vaccination than with one dose in immunocompromised patients^6^, but they are still lower than those in healthy individuals.

In case of kidney transplantation, many living-donor kidney transplantation and ABO-incompatible kidney transplantation have been performed to overcome the donor shortage issue^7^.

The kidney transplant situation is different in Japan from that in other countries, as ABO-incompatible kidney transplants require blood purification procedures, such as plasma exchange and stronger immunosuppression than ABO-compatible transplants. Furthermore, due to a shortage of donors, kidney transplants are performed in some cases when weak positive donor-specific HLA antibodies (DSA) are considered acceptable^7^. Therefore, in addition to ethnic differences, the strength of immunosuppression in kidney transplants may also vary; thus, it is necessary to obtain Japan-specific data. In this study, we measured SARS-CoV-2 antibody titers in post-kidney transplant patients between 3 weeks and 3 months after vaccination to investigate the rates of antibody acquisition.

## Materials and methods

### Patients

Post-kidney transplant recipients who attended Nagoya University Hospital between June and September 2021 were included in this study. Patients who underwent kidney transplantation at other hospitals, but were subsequently attended Nagoya University Hospital, were also included. At the time this study was conducted, two types of vaccines were used in Japan, namely BNT162b2 (Pfizer-BioNTech) and mRNA-1273 (Moderna). Whenever possible, we investigated which of them was used by obtaining the inoculation forms. Patients who failed to receive SARS-CoV-2 mRNA vaccine due to a history of anaphylaxis or allergy were excluded. Patients already on dialysis, post–SARS-CoV-2 infection, and those who failed to provide consent were also excluded. The control group comprised patients (*n* = 8) with chronic kidney disease (CKD) stage G3 or better, including transplant donors and patients with mild IgA nephropathy not requiring steroid therapy. The estimated glomerular filtration rate (eGFR) was calculated using the Japanese equation^8^. Data regarding patient background, past history, comorbidity, medication, and laboratory data were collected. The study was approved by the Institutional Review Board of the Nagoya University Hospital (approval number: 2010–1135 and 2020–0486), and all participants provided written informed consent. All methods were conducted in compliance with the Declaration of Helsinki and the relevant guidelines.

### Measurement of SARS-CoV-2 antibody titers

Serum samples collected on the day of routine medical checkups from 3 weeks to 3 months after two doses of vaccination were used for the measurement. A completely automated immunoassay analyzer (Alinity i; Abbott Laboratories, IL, USA) was used as the analytical instrument. SARS-CoV-2 IgG II Quant Reagent Kit (Lot; 28531FN00, Abbott Japan Co., Ltd.) was used for the SARS-CoV-2 IgG antibody assay. The antibody-positive cutoff was set as at least 50 AU/mL according to the previous reports^9,10^.

### Statistical analysis

Baseline characteristics are presented descriptively. They were tested using the Mann–Whitney U test and χ^2^ test. Univariate and multivariate logistic regression analyses were used to evaluate the rates of antibody acquisition. Differences were considered statistically significant at *P* < 0.05. The R software (R Foundation for Statistical Computing, Vienna, Austria, http://www.R-project.org/) was used to perform all statistical analyses in this study^11^. We also used gglot2 package (https://ggplot2.tidyverse.org.) to draw violin plots^12^.

### Informed consent

Informed consent was obtained from all individual participants included in the study.

## Results

### Patient background and antibody acquisition rates

**Figure 1:**
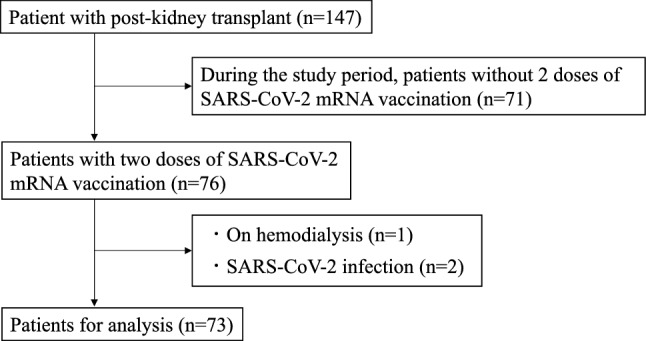
Flow chart showing the process of patient registration.

**Figure 2:**
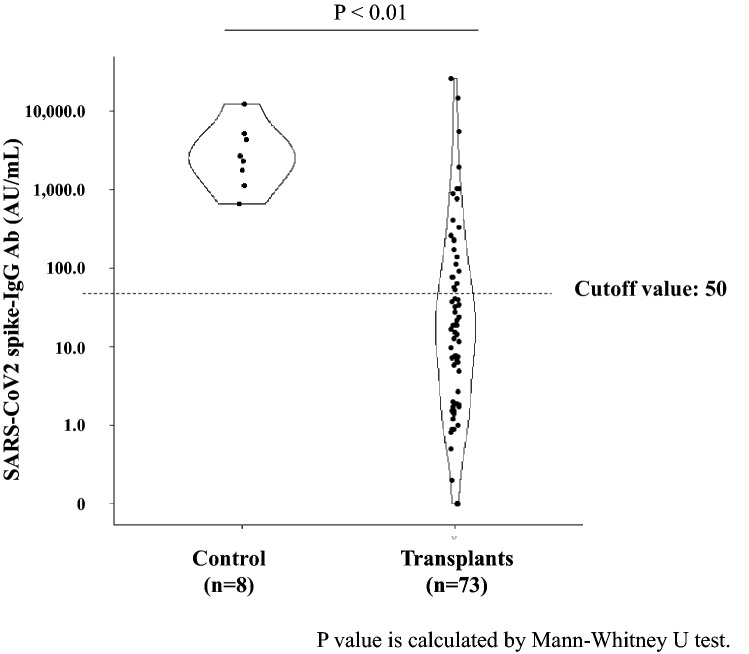
Quantification of antibody titers in participants in the post-kidney transplantation and control groups. The dots indicate the values of SARS-CoV2 spike-IgG Antibodies. The dotted line indicates the cutoff value.

We investigated 73 transplant recipients (45 men and 28 women) who received two doses of SARS-CoV-2 mRNA vaccine (Fig. 1). The baseline characteristics of the patients are shown in Table 1. The median age at the time of vaccination was 61 years (interquartile range [IQR], 50–69 years), and the median age at the time of transplantation was 53 years (IQR, 41–61 years). The median duration between transplantation and vaccination was 74 months (IQR, 30–131 months). The median body mass index (BMI) was 22.5 (IQR, 21.0–25.7). Chronic glomerulonephritis (CGN) was the most common primary kidney disease (35.6%), followed by diabetic nephropathy (DMN) (13.7%), and hereditary disease (12.3%). The coexisting diseases were diabetes mellitus (26.0%) and hypertension (68.5%). Living-donor kidney transplants showed a high rate (91.8%). Preemptive kidney transplantation was performed in 31 patients (42.5%); and of 42 patients who underwent renal replacement therapy (RRT), 32 underwent hemodialysis (HD) and 10 underwent peritoneal dialysis (PD) as pre-transplant RRT. The median pre-transplant RRT period in patients who underwent HD and PD was 23 (IQR, 9–76) months. The blood test immediately before vaccination was 6.7 × 10^3^/μl (IQR, 5.4–7.7) for white blood cells and 1.6 × 10^3^/μl (IQR, 1.2–2.1) for lymphocytes. The immunosuppressive drugs administered were prednisolone in all cases, tacrolimus in 89.0%, cyclosporine in 9.6%, and mofetil mycophenolate (MMF) in 97.3%. Twenty-three (31.5%) patients in the post-kidney transplant group tested positive for acquired antibodies that was a very low percentage than in control group (Fig. 2). Moreover, antibody titer was low in patients who showed acquired antibodies. None of the patients failed to receive the second vaccine dose due to adverse reactions, such as anaphylaxis.

**Table 1:** Clinical characteristics of study participants (n = 73).

Age at the time of vaccination, year, median [IQR]	61 [50, 69]
Age at the time of transplantation, year, median [IQR]	53 [41, 61]
Male, n (%)	45 (61.6)
Height, cm, median [IQR]	163.0 [156.2, 169.0]
Weight, kg, median [IQR]	59.7 [52.4, 70.5]
BMI, kg/m^2^, median [IQR]	22.5 [21.0, 25.7]
eGFR, mL/min/1.73m^2^, median [IQR]	43.2 [34.7, 51.5]
The duration between transplantation and vaccination, month, median [IQR]	74 [30, 131]
The duration from vaccination to antibody assay, day, median [IQR]	41 [31, 53]
**Primary kidney diseases, n (%)**	
Chronic glomerulonephritis	26 (35.6)
Diabetic nephropathy	10 (13.7)
Hereditary disease	9 (12.3)
Nephrosclerosis	3 (4.1)
Focal segmental glomerulosclerosis	3 (4.1)
**Coexisting disease, n (%)**	
Diabetes mellitus	19 (26.0)
Hypertension	50 (68.5)
**Types of transplants, n (%)**	
Deceased donor kidney transplants	6 (8.2)
Living-donor kidney transplants	67 (91.8)
ABO-incompatible kidney transplants	24 (32.9)
Preemptive kidney transplantation, n (%)	31 (42.5)
**RRT before transplantation, n (%)**	
Hemodialysis	32 (43.8)
Peritoneal dialysis	10 (13.7)
Pre-transplant RRT period, month, median [IQR]	23 [9,76]
White blood cell counts immediately prior to vaccination, 10^3^/μl, [IQR]	6.7 [5.4, 7.7]
Lymphocyte counts immediately prior to vaccination, 10^3^/μl, [IQR]	1.6 [1.2, 2.1]
**Types of immunosuppressive drugs, n (%)**	
Prednisolone	73 (100.0)
Mycophenolate mofetil	71 (97.3)
Tacrolimus	65 (89.0)
Cyclosporine	7 (9.6)
**Dose of immunosuppressive drugs, mg/day, median [IQR]**	
Prednisolone	5.0 [5.0, 5.0]
Mycophenolate mofetil	1000 [750, 1250]
Tacrolimus	3.0 [2.0, 4.0]
Cyclosporine	100 [90, 105]
**Types of SARS-CoV-2 vaccine, n (%)**	
Pfizer-BioNTech	52 (71.2)
Moderna	13 (17.8)
Unknown	8 (11.0)

*IQR*, interquartile range; *BMI*, body mass index; *eGFR*, estimated glomerular filtration rate; RRT, Renal replacement therapy.

### Comparison between antibody-acquired and non-acquired groups

**Figure 3:**
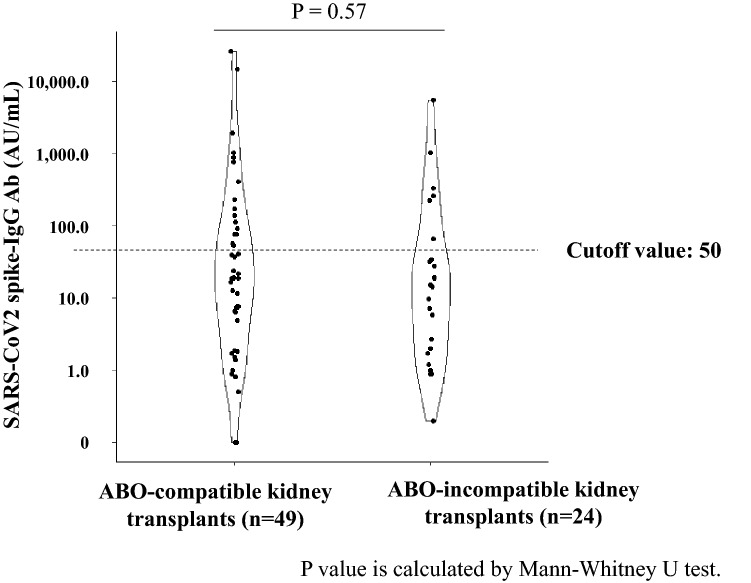
Quantification of antibody titers in ABO-compatible and ABO-incompatible kidney transplant recipients. The dots indicate the values of SARS-CoV2 spike-IgG Antibodies. The dotted line indicates the cutoff value.

Further, we compared the characteristics in the antibody-acquired group with those in non-acquired group of patients who underwent post-kidney transplantation. The characteristics of the patients in each group are presented in Table 2. Patients’ age at transplantation, BMI, eGFR, duration between transplantation and vaccination, and lymphocyte counts immediately prior to vaccination were significantly different between the groups. For instance, in the antibody-acquired group, the shortest duration between transplantation and vaccination was 16 months. There was no significant difference in the antibody acquisition rate between ABO-incompatible and ABO-compatible kidney transplant recipients (Fig. 3).

**Table 2:** Comparison between antibody-acquired and non-acquired groups.

	Antibody positive (n = 23)	Antibody negative (n = 50)	*P* value
Age at the time of vaccination, year, median [IQR]	59 [49, 69]	64 [50, 69]	0.392
Age at the time of transplantation, year, median [IQR]	50 [37, 55]	56 [45, 62]	< 0.05
Male, n (%)	16 (69.6)	29 (58.0)	0.493
Height, cm, median [IQR]	165.8 [160.1, 169.6]	162.0 [154.8, 168.9]	0.269
Weight, kg, median [IQR]	64.2 [57.2, 78.2]	57.7 [51.9, 68.6]	< 0.05
BMI, kg/m^2^, median [IQR]	23.7 [22.2, 27.2]	22.2 [20.6, 24.6]	< 0.05
eGFR, mL/min/1.73m^2^, median [IQR]	47.4 [39.4, 54.4]	41.1 [30.4, 49.3]	< 0.05
The duration between transplantation and vaccination, month, median [IQR]	81 [48, 160]	66 [23, 119]	< 0.05
The duration from vaccination to antibody assay, day, median [IQR]	44 [32, 58]	40 [30, 52]	0.419
**Primary kidney diseases, n (%)**			0.494
Chronic glomerulonephritis	11 (47.8)	15 (30.0)	
Diabetic nephropathy	2 (8.7)	8 (16.0)	
Hereditary disease	1 (4.3)	8 (16.0)	
Nephrosclerosis	1 (4.3)	2 (4.0)	
Focal segmental glomerulosclerosis	1 (4.3)	2 (4.0)	
**Coexisting disease, n (%)**			
Diabetes mellitus	7 (30.4)	15 (24.0)	0.768
Hypertension	13 (56.5)	37 (74.0)	0.222
**Types of transplants, n (%)**			
Deceased donor kidney transplants	2 (8.7)	4 (8.0)	1.000
Living-donor kidney transplants	21 (91.3)	46 (92.0)	1.000
ABO-incompatible kidney transplants	6 (26.1)	18 (36.0)	0.569
Preemptive kidney transplant, n (%)	7 (30.4)	23 (46.0)	0.317
White blood cell counts immediately prior to vaccination, 10^3^/μl, [IQR]	7.2 [5.9, 8.3]	6.5 [5.3, 7.5]	0.121
Lymphocyte counts immediately prior to vaccination, 10^3^/μl, [IQR]	1.9 [1.6, 2.2]	1.4 [1.1, 2.1]	< 0.05
**Types of immunosuppressive drugs, n (%)**			
Mycophenolate mofetil	22 (95.7)	49 (98.0)	1.000
Tacrolimus	18 (78.3)	47 (94.0)	0.110
Cyclosporine	4 (17.4)	3 (6.0)	0.268
**Dose of immunosuppressive drugs, mg/day, median [IQR]**			
Prednisolone	5.0 [5.0, 5.0]	5.0 [5.0, 5.0]	0.148
Mycophenolate mofetil	1000 [750, 1250]	1000 [750, 1000]	0.607
Tacrolimus	2.8 [2.0, 3.8]	3.5 [2.0, 4.0]	0.266
Cyclosporine	90 [75, 100]	110 [105, 130]	0.099
**Types of SARS-CoV-2 vaccine, n (%)**			< 0.05
Pfizer-BioNTech	14	38	
Moderna	8	5	
Unknown	1	7	

*IQR*, interquartile range; *BMI*, body mass index; *eGFR*, estimated glomerular filtration rate.

### Examination of factors associated with antibody acquisition

**Figure 4:**
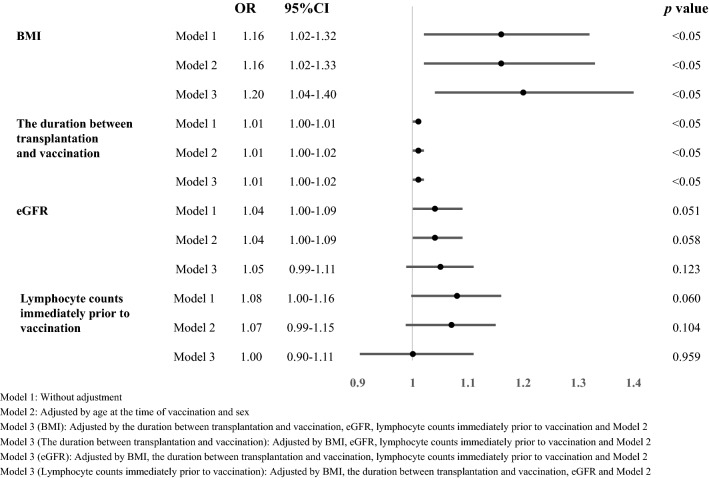
Clinical factors associated with low antibody acquisition rates of post-kidney transplant patients. Odds ratios for lymphocyte counts are shown per 100 cells/uL. OR, odds ratio; CI, confidence interval; BMI, body mass index; eGFR, estimated glomerular filtration rate.

Next, we performed a logistic regression analysis to examine the factors involved in antibody acquisition. Factors found significant in previous analysis (Table 2) were considered in this analysis, by excluding multicollinearity. The analyses suggested that BMI and duration between transplantation and vaccination significantly correlated with antibody acquisition rates, even after adjustment for various factors (Fig. 4).

Additionally, we attempted to confirm the type of vaccine (BNT162b2, Pfizer-BioNTech or mRNA-1273, Moderna) by obtaining details with an inoculation form, but failed to determine all of patients. In patients for whom the vaccine type was known, administration of mRNA-1273 vaccine (Moderna) correlated with a higher rate of antibody acquisition than BNT162b2 (Pfizer-BioNTech) (Fig. S1).

### Regression analysis between antibody-titers and the duration from vaccination to measurement

We measured antibody titers from 3 weeks to 3 months after two doses of vaccination, due to the timing of outpatient visits and vaccination. To investigate the possibility of antibody titer decline over time, we assessed for a correlation between the number of days from second vaccination to antibody titer measurement and the antibody titer values. Table 3 shows the results. No relationship was found between the number of days from vaccination to measurement and antibody titer.

**Table 3:** Regression analysis between antibody-titers and the duration from vaccination to measurement.

	β	95% CI-LL	95% CI-UL	t	*P* value
Model 1	0.0063	−0.0120	0.0245	0.6832	0.4967
Model 2	0.0141	−0.0063	0.0345	1.3758	0.1733
Model 3	0.0073	−0.0104	0.0250	0.8233	0.4133

Model 1: Without adjustment. Model 2: Adjusted by age at the time of vaccination and sex. Model 3: Adjusted by BMI, the duration between transplantation and vaccination, eGFR, lymphocyte counts immediately prior to vaccination and Model 2.

95% *CI*; confidence interval; *LL* lower limit. *UL* upper limit.

## Discussion

The study demonstrated that the rate of antibody acquisition in post-kidney transplant patients was extremely low. Moreover, the study indicated that a significant proportion of post-kidney transplant patients may remain at risk for COVID-19 even after two doses of the mRNA vaccination. A lower BMI and shorter duration between transplantation and vaccination correlated with lower rate of antibody acquisition. Additionally, despite incomplete data, the analyses indicated that there may be differences in antibody acquisition rates depending on the vaccine type.

The rates of antibody acquisition in immunocompromised patients, such as those after organ transplantation, reported in previous studies^6,13^ are higher than those found in the present study. This may be due to the high intensity of immunosuppression in Japan, where many ABO-incompatible transplants are performed. However, the results of Fig. 3 did not support this hypothesis. The lack of significant difference in antibody acquisition between ABO-incompatible and ABO-compatible transplants in the present results may be due to the fact that few patients were vaccinated immediately after transplantation, which has different immunosuppressive strengths. Taking the results into consideration, the low rate of antibody acquisition may be strongly influenced by other factors. Patients in the present study tended to be older and have a lower BMI than that observed in studies from other countries. Evidence suggests that people with a low BMI are more vulnerable to infections^14^, and underweight people tend to have a weakened immunity. The BMI of patients in this study was not extremely low, but it could be lower than that in patients in other countries; therefore, the antibody acquisition rates may have been lower than those in other countries. The duration between transplantation and vaccination remains controversial. In the antibody-acquired group, the shortest duration between transplantation and vaccination was 16 months. We hypothesized that patients are less likely to acquire antibodies immediately after transplantation even if they are vaccinated; thus, extending the duration between transplantation and vaccination may have become a trend and contributed to bias. The antibody acquisition rate may drop considerably for approximately one year after transplantation, although it is difficult to confirm this based on the small sample size in the present study. However, it is ethically incorrect that patients who have undergone transplantation and are receiving strong immunosuppressive therapy be excluded from vaccination for one year. Furthermore, evidence suggests that people who received the third booster vaccine dose have higher antibody titers than those who received only the second dose, which is also true for organ transplant patients^15,16^. Therefore, it is preferable to administer the third dose for infection control, especially in patients with weakened immune systems, such as those immediately after transplantation. Additionally, patients receiving MMF as immunosuppressant have difficulty in acquiring antibodies^6^. In this study, we failed to examine the relationship between immunosuppressive drugs, especially with or without MMF administration, and antibody acquisition rates as a majority of the patients were administering MMF. However, there was no significant difference in MMF dose between the two groups. We observed a difference in eGFR between the antibody-acquired and non-acquired groups, although it was not significant. It has been reported that renal failure is associated with decreased immunity^17^. Therefore, patients undergoing strong immunosuppressive therapy for graft rejection or other reasons, and with impaired renal function should be especially careful.

In the present study, patients vaccinated with mRNA-1273 (Moderna) had a higher rate of antibody acquisition than those vaccinated with BNT162b2 (Pfizer-BioNTech). This may be attributed to differences in the amounts of chemicals contained in the products. Studies suggest that the frequency of side effects varies with the vaccine type^18,19^, and allergic reactions may be suppressed in immunosuppressed patients. None of the patients in the present study canceled the second vaccine dose due to allergic reactions. Thus, considering these factors, interventions to improve vaccine response, such as vaccine type, timing of vaccination, additional booster dose administration, and adjustment of immunosuppressive drugs, need to be further investigated in post-kidney transplant patients.

The present study has a few limitations. First, it was a retrospective study. Second, the study had a limited number of participants and was conducted at a single center. Third, some of the participants failed to report the type of vaccine they had received. However, despite the limitation, it is extremely important to show the antibody acquisition rate after two doses of vaccinations in kidney transplant recipients because it provides basic information for clinical actions, such as infection control and the third vaccination.

## Conclusion

The antibody response to two doses of SARS-CoV-2 vaccine in Japanese kidney transplant recipients was lower than that in healthy subjects. Thus, post-kidney transplant patients may remain at risk of infection with only two doses of vaccination, and it is necessary to closely monitor infection control.

## Supplementary Information

Supplementary Information 1.

Supplementary Information 2.

## Data Availability

The datasets generated and analyzed during the current study are not publicly available because consent has not been obtained to make them available online to an unspecified number of people, but they are available from the corresponding author on reasonable request.
