# Supplementary material for: Antibody response to double SARS-CoV-2 mRNA vaccination in Japanese kidney transplant recipients
Source: Sci Rep. 2022 Apr 27;12:6850. doi: 10.1038/s41598-022-10510-7 (PMC9043506; doi:10.1038/s41598-022-10510-7)
Supplement: Supplementary file 1 — Supplementary Information 1. [file 41598_2022_10510_MOESM1_ESM.pptx]

## Slide 1
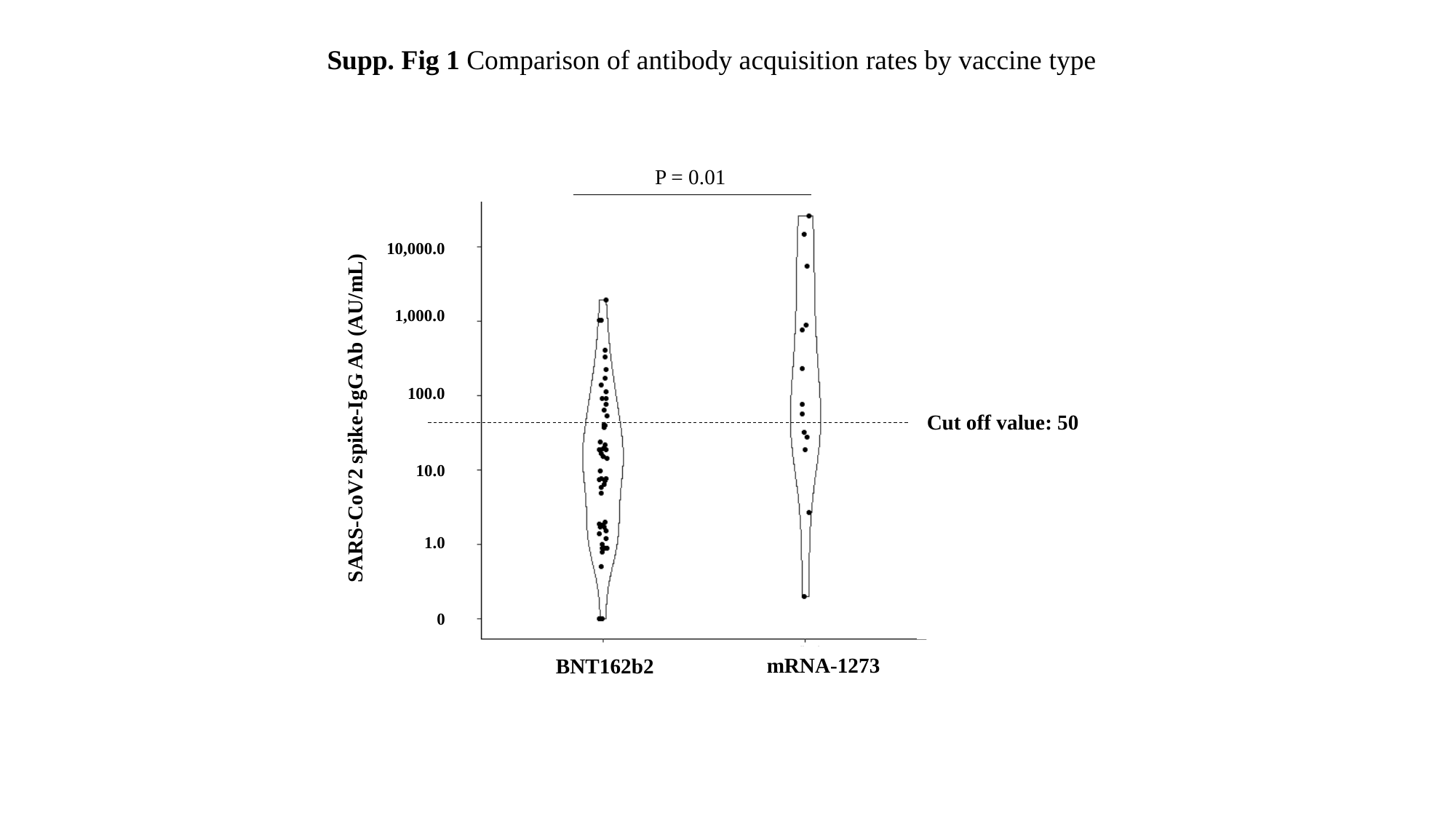

Supp. Fig 1 Comparison of antibody acquisition rates by vaccine type
P = 0.01
10,000.0
1,000.0
100.0
Cut off value: 50
10.0
1.0
0
BNT162b2
mRNA-1273
SARS-CoV2 spike-IgG Ab (AU/mL)
