# Supplementary material for: Antibody response to double SARS-CoV-2 mRNA vaccination in Japanese kidney transplant recipients
Source: Sci Rep. 2022 Apr 27;12:6850. doi: 10.1038/s41598-022-10510-7 (PMC9043506; doi:10.1038/s41598-022-10510-7)
Supplement: Supplementary file 2 — Supplementary Information 2. [file 41598_2022_10510_MOESM2_ESM.docx]

**Fig. S1** Comparison of antibody acquisition rates by vaccine type

The dots indicate the values of SARS-CoV2 spike-IgG Antibodies. The dotted line indicates the cutoff value.
